# Clinical-Epidemiology Aspect of Inpatients With Moderate or Severe COVID-19 in a Brazilian Macroregion: Disease and Countermeasures

**DOI:** 10.3389/fcimb.2022.899702

**Published:** 2022-05-20

**Authors:** Bruna Raphaela Oliveira Silva, Wellington Francisco Rodrigues, Daniela Gomes Pires Abadia, Djalma A. Alves da Silva, Leonardo E. Andrade e Silva, Chamberttan S. Desidério, Thais Soares Farnesi-de-Assunção, Juliana C. Costa-Madeira, Rafaela M. Barbosa, Anna V. Bernardes e Borges, Andrezza C. C. Hortolani Cunha, Loren Q. Pereira, Fernanda R. Helmo, Marcela Rezende Lemes, Laís M. Barbosa, Rafael O. Trevisan, Malu Mateus Santos Obata, Giovanna F. Bueno, Fabiano V. Mundim, Ana Carolina M. Oliveira-Scussel, Ivan B. Monteiro, Yulsef M. Ferreira, Guilherme H. Machado, Kennio Ferreira-Paim, Hélio Moraes-Souza, Marcos Vinicius da Silva, Virmondes Rodrigues Júnior, Carlo José Freire Oliveira

**Affiliations:** ^1^ Department of Immunology, Microbiology and Parasitology, Federal University of Triângulo Mineiro, Uberaba, Brazil; ^2^ Postgraduate Program in Physiological Sciences, Federal University of Triângulo Mineiro, Uberaba, Brazil; ^3^ Laboratory of Hematological Research of the Federal University of Triângulo Mineiro and Regional Blood Center of Uberaba - Hemominas Foundation, Uberaba, Brazil; ^4^ UNIMED São Domingos Hospital, Uberaba, MG, Brazil. José Alencar Gomes da Silva Regional Hospital, Uberaba, Brazil; ^5^ José Alencar Gomes da Silva Regional Hospital, Uberaba, Brazil; ^6^ Mário Palmério University Hospital, Uberaba, Brazil

**Keywords:** epidemiology, COVID-19, inpatients, treatment, Brazil

## Abstract

COVID-19, also known as coronavirus disease 2019, is an infectious viral disease caused by SARS-CoV-2, a novel coronavirus. Since its emergence, its epidemiology has been explored; however, for some regions of the world, COVID-19’s behavior, incidence, and impact remain unclear. In continental nations like Brazil, this lack of knowledge results in nonuniform control, prevention, and treatment measures, which can be controversial in some locations. This study aimed to describe the epidemiological profile of patients with COVID-19 in the macroregion of Triângulo Sul in the state of Minas Gerais (MG), Brazil. Between March 25 and October 21, 2020, data were collected and statistically analyzed from 395 hospitalized patients in the city of Uberaba, MG, suspected to have moderate or severe forms of the disease. Of the 395 suspected cases, 82% were confirmed to be positive for COVID-19. The mean age of positive patients was 58.4 years, and 60.76% were male. Following these patients throughout their hospitalization, a mortality rate of 31.3% was observed. In the population positive for COVID-19, the risk of death increased by 4% for each year of the patient’s age. Likewise, the older the patient, the longer their hospitalization and the higher the risk of developing acute respiratory failure. Among the treatments tested in patients, heparin was associated with protection against mortality, and the absence of anticoagulant use was linked to a more than six times greater risk of death. Finally, comorbidities in patients with COVID-19 were positively correlated with increased hospitalization time. In summary, this study revealed that age, presence of comorbidities, length of hospitalization, and drug treatment considerably altered COVID-19’s lethality. To understand infection rates and the factors involved in COVID-19’s lethality, knowledge of the local epidemiology is necessary.

## Introduction

Despite understanding the health-damaging potential of coronaviruses, signaled in 2002 and 2003 by Severe Acute Respiratory Syndrome Coronavirus (SARS-CoV), which caused severe acute respiratory syndrome, and Middle East Respiratory Syndrome Coronavirus (MERS-CoV), which caused severe acute respiratory syndrome, and MERS-CoV, which caused Middle East respiratory syndrome, coronavirus infections these have relatively low social, economic, and health impacts (Ganesh et al., 2021; Zhou et al., 2021). Unlike the 2002 and 2003 coronaviruses, on December 31, 2019, Chinese health authorities reported to the World Health Organization numerous cases of respiratory infections caused by a new coronavirus strain initially named 2019-nCoV (Rastogi et al., 2020). After further investigation of the virus, on February 11, 2020, it was renamed SARS-CoV-2, the virus causing coronavirus disease 2019 (COVID-19). The virus quickly spread to East Asia, including South Korea and Japan, followed by New Zealand, and then cases were reported in Europe and the Americas. The first case was reported in Brazil on February 19, 2020 (Cavalcante et al., 2020).

Currently, SARS-CoV-2 is present globally, and multiple variants were identified and characterized, causing pathologies beyond initially reported lung and airway problems (Hemmer et al., 2021; Rahimi Pordanjani et al., 2021). In practical terms, COVID-19 has become a global threat, and measures of prophylaxis, treatment, and vaccination are continuously being explored to improve the fight against this disease that is spreading faster daily.

In general, the clinical manifestations of COVID-19 in 80% of cases include fever, cough, myalgia, fatigue, anorexia, nasal congestion, headache, anosmia, respiratory symptoms, dyspnea, and gastrointestinal manifestations (Jogalekar et al., 2020; World Health Organization, 2020). Less common clinical symptoms include encephalopathy, delirium, agitation, meningoencephalitis, anxiety and depression, pulmonary embolism, acute coronary syndrome, and stroke (World Health Organization, 2020). Data from the first year of the pandemic report that 6–41% (with an average estimate of 16%) of those infected are asymptomatic (Byambasuren et al., 2020). Among symptomatic cases, approximately 40% of patients display mild clinical conditions, 40% of patients have moderate disease, about 15% of patients with severe disease require ventilatory support, and 5% of patients develop critical conditions such as sepsis, shock, thromboembolism, and multiple organ failure (Jogalekar et al., 2020).

Global data emphasize that mortality rates vary according to factors, including age group, treatment, pre-existing clinical conditions, and virulence of the strain and its variants (Halaji et al., 2021; Cascella et al., 2022). At this point, the severity of symptoms is lower in children than in adults, especially the elderly population (Jogalekar et al., 2020). In addition to infection and mortality factors, the pandemic death rates are associated with the lack of financial and structural support in the affected countries and insufficient public policies that contradict the health measures required for reducing mortality in the population (Ataguba and Ataguba, 2020; Bambra et al., 2020). The absence of gold standard treatment for COVID-19 and the unequal distribution of vaccines in different countries are situations that must be considered when discussing the fight against COVID-19. In addition to drug and preventive treatment, the availability of vaccines, medical resources, and other health supports are key (Ahn et al., 2020; Ciotti et al., 2020; Lippi and Plebani, 2020).

Brazil is a classic example where direct and indirect variables have a strong impact on the disease’s epidemiology. In the first and second years of the pandemic, there were several outbreaks, uncontrolled dissemination, lack of resources, and the use of repurposed drugs with proven ineffectiveness, among many other factors impacting the epidemiology of the disease (Dal Poz et al., 2021; Hallal and Victora, 2021). Despite the various studies carried out to date, few studies evaluated the epidemiology and outcomes of hospitalized patients, especially in regions farther from capital cities, including those that were rural or less developed. In this study, we describe the epidemiological profile of patients positive for COVID-19 in the macroregion of southern triangle, MG, Brazil. This study characterizes the epidemiological aspects of hospitalized patients treated in the municipality of Uberaba, MG, a city that treated approximately 800 patients from the macroregion since the beginning of 2020.

## Materials and Methods

### Study Patients

Patients selected for the present study were hospitalized with a flu-like illness and suspected to have COVID-19 in three hospitals in the city of Uberaba, MG, Brazil. These hospitals were responsible for COVID-19 patients from across the southern triangle macroregion (Mário Palmério University Hospital, José Alencar Gomes da Silva Regional Hospital, and Unimed São Domingos Hospital). The southern triangle region (Triângulo Mineiro) covers 27 municipalities with a population of approximately 800,000 inhabitants and is predominantly urban (91.67%) (IBGE – INSTITUTO BRASILEIRO DE GEOGRAFIA E ESTATÍSTICA, 2019). Patients admitted to a nursery or intensive care unit with suspected COVID-19 were included in the study.

### Patients’ Diagnosis

COVID-19 diagnosis was confirmed by qRT-PCR analysis of nasal swabs, nasopharyngeal aspirate, or lower respiratory secretion samples (sputum, tracheal lavage, or bronchoalveolar lavage) collected before or during hospitalization. Patients negative for COVID-19, classified as a flu-like syndrome, were included as negative controls.

### Ethics

The present study was approved by the Research Ethics Committee of the Hospital de Clínicas, Universidade Federal do Triângulo Mineiro (HC-UFTM) (approval number: 3.957.676), and conducted according to principles of the Declaration of Helsinki. Between March 25 and October 21, 2020, patients who agreed to participate in the study signed an informed consent form, while patients who could not sign were included after a legal guardian’s signature, authorizing the analysis of their medical records, laboratory results, and imaging data.

### Study Design

This was a quantitative descriptive study in which information including patient age, inpatient hospital, SARS-CoV-2 test results, symptom duration prior to hospitalization, length of hospital stay, sex, mortality, pre-existing comorbidity diagnosis, and administered medications were extracted from the medical records and entered into an electronic datasheet (**Table 1**). It was not possible to establish the protocols for drug intervention individually, so the information for the use of drugs was categorized with two possible outcomes, used or not used. Entries that did not include any of the variables selected for the study were excluded from this analysis.

**Table 1:** List of drugs administered to patients before and/or after hospitalization*.

Drug	Active substance
Antimalarial/antirheumatic	Hydroxychloroquine (400 mg/day initially and then reduced to 200 mg/day).
Antibiotics	Amikacin (7.5 mg/kg every 12 hours or 5 mg/kg every 8 hours), Ampicillin Sulfate + Sulbactam (1.5g to 12g per day in divided doses every 6 or 8 hours), Azithromycin (500 mg daily for 3 to 5 days), Cefepime (1-2g every 8-12 hours), Ceftriaxone (1-2 g every 24 hours), Cefuroxime (750 mg three times a day), Clarithromycin (250 mg every 12 hours), Clindamycin (600 – 1,800 mg, divided into 2, 3 or 4 equal doses), Moxifloxacin Hydrochloride (400 mg once a day), Gentamicin (160 mg as a single daily dose or 80 mg every 12 hours), Imepenem (1 to 2 g, given in 3 or 4 divided doses), Levofloxacin (500 mg as a single daily dose for 7 to 10 days), Linezolid (600 mg every 12 hours for 14 to 28 days), Meropenem (1.5 to 6.0 g daily, divided into three administrations), Metronidazole (400 mg three times a day for 7 days), Oxacillin (1 g or more every 4 to 6 hours), Piperacycline + Tazobactam (12 g piperacillin/1.5 g tazobactam divided into doses every 6 or 8 hours), Polymyxin B (25.000 a 30.000 UI/kg/dia), Sulfamethoxazole + Trimethoprim (800mg + 160mg twice daily), Teicoplanin (3 doses of 400 mg every 12 hours), Vancomycin (2g, divided into 500mg every 6 hours or 1g every 12 hours), others.
Anticoagulants	Heparin (20,000 to 30,000 IU in 1 liter of solution, 15 drops per minute, for 24 hours), Enoxaparin (1.5 mg/kg once a day or 1 mg/kg twice a day), Apixaban (10 mg twice daily), Enoxaparin sodium (1.5 mg/kg once daily or 1 mg/kg twice daily).
Antifungals	Clotrimazole (10 mg/g, 2 times daily for 2 to 4 weeks), Fluconazole (100 mg as a single daily dose for at least 2 weeks), Micafungin (150mg/day).
Antifungal, antiprotozoal	Amphotericin B (the dose should not exceed 1.5 mg/kg/day).
Anti-inflammatories	Dexamethasone (0.75 to 15 mg per day), Hydrocortisone (100 to 500 mg; repeat, if necessary, every 2 to 6 hours), Methylprednisolone (30mg/kg, every 4 to 6 hours for up to 48 hours), Prednisolone (5 to 60 mg per day).
Antiparasitic	Albendazole (400 mg/day, single dose), Ivermectin (150 mcg/kg to 200 mcg/kg per day).
Antiviral	Oseltamivir (75 mg twice a day for five days).

*The drugs reported on the table were extracted from the patients’ medical records. The concentrations shown represent the recommended doses. Information for treatments prior to admission was reported by patients during the anamnesis (it was not possible to detect the therapeutic scheme for each patient).

### Statistical Analyses

For statistical tests, data were tabulated in Microsoft Excel and analyzed using the IBM SPSS Statistics 21 and Jamovi 1.6.15 software (Pallant, 2020). For descriptive analyses, absolute (n) or relative frequencies with 95% confidence intervals were used. The chi-squared test was used to verify the associations of proportions in the different categories of the analyzed variables. A binomial logistic regression model for prediction and estimation of the evaluated coefficient effects was incorporated for predictive generation in three blocks. The first contained age, sex, length of hospital stay, and presence or absence of comorbidity. The second had positive or negative for acute respiratory failure, renal failure, and anxiety diarrhea. The third block included use of hydroxychloroquine, antibiotics, antifungals, antiparasitics, antivirals, or corticosteroids.

For the model adjustment, the pseudo R^2^ indicated by McFadden’s R^2^, multicollinearity with a tolerance > 80%, and possible outliers, indicated after obtaining Cook’s distance, were considered. The overall hit rate was determined based on accuracy. The Spearman test was used after the normality test (Shapiro-Wilk test) and homoscedasticity (Levene test) to evaluate the effects of the correlations presented. The significance level used for all analyses was 5% (Arango, 2001).

## Results

After collecting, verifying, and validating the data, this study evaluated 395 hospitalized patients with suspected COVID-19 diagnosis. Among the patients, the majority were male (60.76%) and aged ≥ 61 years (p < 0.001, for both categories). The evaluated patients had a minimum age of 14 years and a maximum age of 100 years, with a median of 58.3 years and a mean of 58.40 ± 17.10 years. For one of the 395 individuals evaluated, age was not recorded. COVID-19 diagnosis was confirmed in 82.28% (n = 325) of the hospitalized patients, and 17.72% (n = 70) of inpatients showed negative results. All patients continued their respective hospital standard of care interventions. Respiratory and acute renal failure were present in 18.73% and 12.41% of patients, respectively. In addition, intestinal changes marked by diarrhea were observed in 4.81% of patients (n = 19) (**Table 2**).

**Table 2:** Distribution of hospitalized patients with a suspected COVID-19 diagnosis in the macroregion of the Triângulo Mineiro, Southeast of Brazil by sex, age group, diagnostic confirmation, pulmonary, renal or intestinal disorders, use of drugs, and hospitalization outcome between March and October 2020.

POPULATION CHARACTERISTIC			
SEX	n	%	p Value
Male	240	60.76	<.001
Female	155	39.24	
AGE GROUP - YEARS	n	%	p Value
10 to 20	4	1.02	<.001
21 to 30	16	4.06	
31 to 40	46	11.68	
41 to 50	72	18.27	
51 to 60	85	21.57	
≥61	171	43.40	
TEST FOR COVID-19	n	%	p Value
Negative	70	17.72	<.001
Positive	325	82.28	
ACUTE RESPIRATORY FAILURE	n	%	p Value
Yes	74	18.73	<.001
No	321	81.27	
ACUTE RENAL FAILURE	n	%	p Value
Yes	49	12.41	<.001
No	346	87.59	
DIARRHEA	n	%	p Value
Yes	19	4.81	<.001
No	376	95.19	
HYDROXYCHLOROQUINE	n	%	p Value
Yes	21	5.32	<.001
No	374	94.68	
ANTIBIOTICS	n	%	p Value
Yes	317	80.25	<.001
No	78	19.75	
ANTIFUNGALS	n	%	p Value
Yes	31	7.85	<.001
No	364	92.15	
ANTIPARASITIC	n	%	p Value
Yes	8	2.03	<.001
No	387	97.97	
ANTIVIRAL	n	%	p Value
Yes	21	5.32	<.001
No	374	94.68	
CORTICOSTEROID	n	%	p Value
Yes	309	78.23	<.001
No	86	21.77	
ANTICOAGULANT	n	%	p Value
Yes	325	82.28	<.001
No	70	17.72	
OUTCOME	n	%	p Value
Discharged	266	67.34	<.001
Death	124	31.39	
Not determined	5	1.27	

N, number (absolute frequency); %, percentage (relative frequency). Significance level, 5% (chi-squared test).

In evaluating the frequency of drug interventions, we observed the use of heparin (82.28%; n = 325), antibiotics (80%; n = 317), corticosteroids (78.23%; n = 309), hydroxychloroquine (5.32%; n = 31), antifungals (7.85%; n = 31), antivirals (5.32%; n = 21), and antiparasitics (2.03%; n = 8) in the patients evaluated (**Table 2**). Infection outcomes were assessed based on patient discharge or death reports. The majority (67.34%, n = 266) of the patients were discharged, 31.39% (n = 124) of the patients died, and five patients could not be followed prior to the study’s cutoff. The frequency of death increased to 34.20% for patients positive for COVID-19 (**Table 2**).

**Figure 1:**
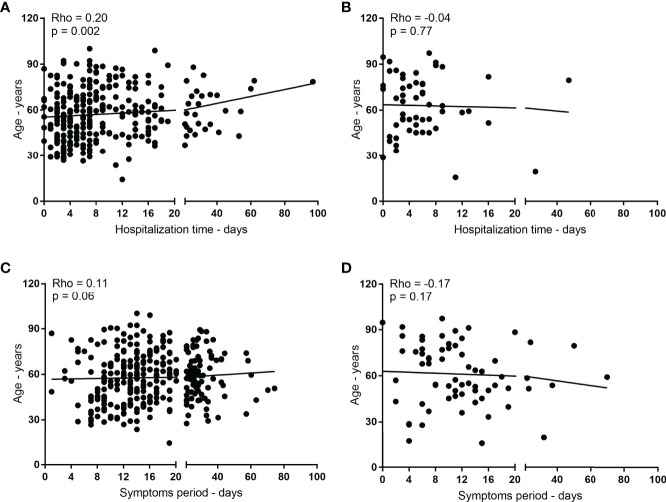
Influence of age on the length of hospitalization and symptomatic period of patients with COVID-19. In **(A, B)**, correlation between age (years) and length of hospitalization (days) for positive and negative patients for COVID-19, respectively. In **(C, D)**, correlation between age (years) and period of symptoms (days) for COVID-19 positive and negative patients, respectively.

Given the overlap in the frequency of senescence in patients, a weak positive and significant correlation was observed between age and hospitalization length of patients positive for COVID-19 (rho = 0.20; **Figure 1A**) but not for those negative for the disease (rho = -0.04; **Figure 1B**). In addition, age was evaluated for correlation with the period of symptoms in patients with COVID-19 (rho = 0.11; **Figure 1C**) or negative (rho = -0.17; **Figure 1D**), but no significant evidence was found.

Patients hospitalized under a diagnostic hypothesis for COVID-19 in the southern triangle macroregion, southeast Brazil, were correlated with their age in years with the length of stay and period of symptoms in days. (A and B) The correlation between age and length of stay of patients positive and negative for COVID-19, respectively. (C and D) The correlation between age and period of symptoms of patients positive and negative for COVID-19, respectively. Spearman’s test was used to verify the correlations. The significance level used was 5%.

Due to the greater number of elderly people hospitalized due to COVID-19, we verified the effect of age on mortality in patients (**Table 2**).

In the evaluated population, whose mean age was 58.40 ± 17.10 years, for each additional year of life, the risk of death increased by 4% (CI = 1.03 to 1.06). Thus, for every 10 years of life, there is a 40% increase in the odds of death (**Table 3**). Sex and the presence of comorbidities (in general) were not factors associated with outcome in the population evaluated. Conversely, each additional day of hospitalization increased the odds of death by 5% (CI = 1.02 to 1.08), with the mean length of stay equaling 10.10 ± 11.10 days, with a minimum value of 0 and a maximum of 97 days (median = 7 days) (**Table 3**). For COVID-19 negative patients with symptoms suggestive of the disease, death was not associated with age, sex, length of stay, or comorbidities (p > 0.05) (**Table 3**). The accuracies observed for the models with the aforementioned variables were 71.50% (positive for COVID-19) and 78.60% (negative patients).

**Table 3:** Binomial logistic regression model to verify the association of the variables age, sex, length of stay, and absence or presence of comorbidities with death in hospitalized patients with suspected COVID-19.

Deaths in patients with COVID-19				
	Odds ratio	Lower	Upper	p Value
Age - Years	1.04	1.03	1.06	< 0.001
Sex (male vs female)	1.08	0.62	1.91	0.779
Length of hospital stay - days	1.05	1.02	1.08	0.002
Comorbidity (present vs absent)	1.53	0.94	2.49	0.09
Negative patients with symptoms similar to COVID-19				
	Odds ratio	Lower	Upper	P Value
Age - Years	1.04	1.00	1.08	0.07
Sex (male vs female)	1.13	0.27	4.78	0.87
Length of hospital stay - days	0.97	0.87	1.10	0.67
Comorbidity (present vs absent)	0.55	0.12	2.46	0.44

vs, versus.

Some of the main aggravating factors associated with severe COVID-19 in hospitalized patients, such as acute respiratory failure, renal failure, and diarrhea, were verified as possible links to the hospitalization outcome (**Table 4**). The presence of acute respiratory failure increased the odds of death by more than 19 times (OR = 19.21; CI = 7.09 to 52.08%) in COVID-19 positive patients. Acute renal failure and diarrhea were not associated with death. For COVID-19 negative, who were symptomatic, there was no significant association with the outcome of the variables described above (**Table 4**). The influence of acute respiratory failure on death resulted in an accuracy of 80.00% in the COVID-19 positive model.

**Table 4:** Binomial logistic regression model to verify the association of the variables: acute respiratory failure, acute renal failure, and diarrhea, in the deaths of hospitalized patients with suspected COVID-19, confirmed or not.

Deaths in patients with COVID-19				
	Odds ratio	Lower	Upper	p Value
Acute respiratory failure				
Yes – No	19.21	7.09	52.08	< .001
Acute renal failure				
Yes – No	2.11	0.67	6.65	0.202
Diarrhea				
Yes – No	0.45	0.12	1.75	0.25
**Initial hypothesis of COVID-19 (negative patients)**				
	Odds ratio	Lower	Upper	p Value
Acute respiratory failure				
Yes – No	2.07	0.44	9.67	0.356
Acute renal failure				
Yes – No	0.68	0.07	6.71	0.739
Diarrhea				
Yes – No	0.00	0.00	Inf	0.992

Inf, infinite. “Yes”, positive for the variable. “No”, negative for the variable.

Considering the variability of intervention protocols, drug treatment was also evaluated for possible associations with the outcome of patients with or without COVID-19 (**Table 5**). Antifungal use was related to a greater than fivefold increase in deaths (CI = 2.32% to 12.49%) in patients positive for COVID-19. In contrast, the use of heparin was associated with protection (OR = 0.37; CI = 0.18 to 0.74%), and the absence of anticoagulant use was linked to an approximately sixfold greater risk of death (OR = 5.93; CI = 1.97% to 17.82%) (**Table 5**). In patients initially diagnosed with COVID-19 but were negative, none of the abovementioned drugs were significantly associated with the outcome (**Table 5**). The accuracy of the model associated with the use of drugs for patients with COVID-19 was 72.70%.

**Table 5:** Binomial logistic regression model to verify the association of variables: use of hydroxychloroquine, antibiotic, antifungal, antiparasitic, antiviral, or heparin, in the deaths of hospitalized patients with a suspected COVID-19 diagnosis.

Deaths in patients with COVID-19				
	Odds ratio	Lower	Upper	p-value
Hydroxychloroquine				
Yes – No	1.85	0.70	4.92	0.215
Antibiotic				
Yes – No	1.67	0.83	3.36	0.147
Antifungal				
Yes – No	5.38	2.32	12.49	< .001
Antiparasitics				
Yes – No	0.59	0.10	3.64	0.571
Hydroxy/antiparasitics				
Yes – No	1.17	0.49	2.77	0.720
Antiviral				
Yes – No	1.46	0.51	4.15	0.478
Anticoagulant				
Yes – No	0.37	0.18	0.74	0.005
**Initial hypothesis of COVID-19 (negative patients)**				
	Odds ratio	Lower	Upper	p-value
Hydroxychloroquine				
Yes – No	0.00	0.00	Inf	0.995
Antibiotic				
Yes – No	0.75	0.14	4.02	0.733
Antifungal				
Yes – No	0.00	0.00	Inf	0.997
Antiparasitics				
Yes – No	0.00	0.00	Inf	0.997
Hydroxy/antiparasitics				
Yes – No	2.33E-07	0.00	Inf	0.991
Antiviral				
Yes – No	2.01	0.15	27.83	0.602
Anticoagulant				
Yes – No	1.06	0.23	4.99	0.94

Inf, infinite; Hydroxy/antiparasitics, hydroxychloroquine/antiparasitics (the data were grouped).

## Discussion

COVID-19 is a viral disease with devastating effects worldwide. In Brazil, the disease epicenter occurred in 2020, and the lethality rate was high compared to most other affected countries. In Brazil the COVID-19’s epidemiology in each region and state varied and is still not well understood. This study evaluated the epidemiological aspects of COVID-19 in the southern triangle macroregion, a considerably affected region in the country’s interior with structural and public health conditions representative of many regions of Brazil.

Epidemiological analysis of the southern triangle macroregion during 2020 revealed that age, presence of comorbidities, hospitalization length, and drug treatment considerably altered the lethality of the disease. Of the 395 patients with a suspected SARS-CoV-2 infection, 82% were confirmed positive. The mean age of the hospitalized patients was 58.4 years. Of the hospitalized patients, 60.76% were male, and during follow-up, a mortality rate of 31.3% was observed. In the population positive for COVID-19, the odds of death increased by 4% for each year of age in the evaluated patients. Likewise, the older the patient, the longer the hospitalization and the higher the risk of developing acute respiratory failure. Among the treatments tested in patients, heparin was associated with protection, and the absence of anticoagulant treatment was linked to a greater than sixfold risk of death. Finally, the occurrence of comorbidities in patients with COVID-19 was positively correlated with an increase in hospitalization (63% of patients).

First, we demonstrated that most hospitalized patients who tested positive for COVID-19 were elderly men, with a mean age of 58.4 years. The mortality rate in patients positive for COVID-19 was 31.39%. Regarding sex, published data reinforced that men are more likely to be hospitalized, die, or present with different clinical variables characterizing the disease (Gomez et al., 2021; Huang et al., 2021). It should be first noted that women live longer than men in most countries (Barford et al., 2006). Women also have a greater chance of survival in the face of disease, epidemics, or difficult survival situations, such as slavery (Barford et al., 2006; Zarulli et al., 2018). This information suggests that social, environmental, and biological factors are crucial for this resilience in women.

Importantly, in studies on other coronaviruses, including SARS-CoV and MERS-CoV, mortality was also higher in men, as with COVID-19 (Ahrenfeldt et al., 2021). However, unlike COVID-19 positive outcomes and hospitalization, the mortality rate was not affected by sex or presence of patient comorbidities in the present study. Mortality was so common in elderly patients that it may have masked differences in mortality related to sex or presence of comorbidities. Another possibility is that the number of patients in the study was insufficient to measure variation or that the clinical follow-up of comorbidities in the first year of the pandemic was not as controlled or understood as it is today. In summary, these data deserve consideration as they show differences in this macroregion when compare to the literature.

Our data showed that advanced age is a central factor in the hospitalization and mortality of COVID-19 patients, regardless of whether they are male or female. The first point to consider is that the elderly population requires special care. Immunosenescence is a natural factor hindering immune defense mechanisms, and several other biological, environmental, and social factors inherent to aging need to be considered. According to (Iser et al. 2020), of the patients symptomatic for COVID-19, 10% will progress to a serious condition, and 5% will require intensive care. As with virulence, mortality in patients aged > 60 years is higher (Turci et al., 2020), which was indeed observed in this study, where mortality was higher in patients aged > 50 years. Elderly people tend to have alterations in the function and cellular composition of innate and adaptive immunity (immunosenescence), with inadequate functioning of T and B cells and increased cytokine production representing a high risk for complications (Valiathan et al., 2016; Chen et al., 2021). Thus, it is strongly recommended that elderly individuals aged > 60 years, especially those with comorbidities, adopt measures to restrict social contact and that health professionals pay special attention to symptoms indicative of COVID-19 in this group (Uehara, 2020).

COVID-19 is primarily characterized by the presence of pneumonia, which is the most serious symptom of the disease and is characterized by cough, fever, shortness of breath, chest pain, dyspnea, fatigue, and a bilateral diffuse interstitial pattern on chest radiography (Mokhtari et al., 2020). However, adverse effects commonly develop in different organs and tissues of the body, including the liver, blood vessels, intestine, adipose tissue, central nervous system, heart, kidneys, and reproductive system (Lopes-Pacheco et al., 2021). Our findings showed that 18.73%, 12.41%, and 4.81% of patients had respiratory failure, acute renal failure, and diarrhea, respectively. These data indicate that, in addition to the airways, some patients had other organs affected by infection or disease. These data are interesting and demonstrate the importance of a complete and adequate clinical evaluation of changes in different systems and organs beyond the lungs and airways.

Lung tissue, by nature, has several characteristics that make it the initial focus of infection, replication, and transmission to other individuals. Once inhaled through the airways, viruses easily disperse in the lung tissue that is large and highly vascularized and has a high expression of the angiotensin-converting enzyme-2 protein, especially in endothelial and other lung cells (Hamming et al., 2004; Albini et al., 2020). This explains why some comorbidities make patients more susceptible to infections and diseases. For example, cardiovascular diseases, hypertension, and diabetes increase angiotensin-converting enzyme-2 expression in multiple cell types (Drucker, 2021).

Among the most urgent challenges in the fight against COVID-19, the infection of individuals with comorbidities is of great concern. Findings from around the world clearly demonstrate that individuals with comorbidities are more prone to severe forms of the disease, especially patients with obesity, diabetes, cardiovascular disease, hypertension, or cancer (Ejaz et al., 2020). In fact, the presence of comorbidities is an indication of increased risk during hospitalization (Bastos et al., 2020; Zhou et al., 2020), which can greatly impact the course of the epidemic. Of the patients enrolled during the study period, approximately 63% had comorbidities, including arterial hypertension, asthma, and diabetes.

While the mortality rate observed in Triângulo Mineiro (includes the southern triangle macroregion) between 2014 and 2019 due to respiratory diseases was approximately 8% (Dias et al., 2020), during the period of this study, SARS-CoV-2 infection resulted in a mortality of almost 35% of hospitalized patients. The high prevalence of patients with comorbidities in the study region, combined with the crisis, a novel virus, and new drugs and intervention protocols to control virus spread and severity, underscored the need for epidemiological analysis.

Among the most controversial issues surrounding the COVID-19 pandemic, treatment option discussion was the most contentious and remains controversial. Epidemiological data from the southern triangle macroregion showed the population used different medications for the treatment of COVID-19. Some were shown to have no protective effects, others are still being studied, and others are considered partially beneficial in the fight against COVID-19. Patients hospitalized in southern triangle macroregion were treated with hydroxychloroquine, antibiotics, antifungals, antiparasitics, antivirals, corticosteroids, or anticoagulants.

Importantly, our data showed marked differences in patient outcomes, depending on the drugs used. During our study, many patients were undergoing treatment with repurposed drugs (some of them without a doctor’s prescription) prior to any definitive evidence of efficacy. Antifungal use was related to a greater than fivefold increase in death, and anticoagulant use was associated with protection from death.

Although there is no consensus recommending anticoagulant treatment for COVID-19, especially because the studies are still observational (*in vitro* and *in vivo*) or include few participants (Billett et al., 2020; Medeiros et al., 2020; Ramos and Ota-Arakaki, 2020), its absence as a prophylactic measure considerably increased the number of deaths in the patients evaluated in the present study. The rationale for anticoagulants use is based on the fact that COVID-19 is a severe acute respiratory syndrome that may induce inflammation of the vascular system and increase coagulation with consequent vasculitis and thrombosis (Sarkar et al., 2021). The findings of our study suggest benefits to the patient with anticoagulant use, and more robust studies and randomized controlled trials are needed to confirm this finding.

The use of antifungals, antimalarials, and other drugs that inhibit autophagy, such as rapamycin, chloroquine and hydroxychloroquine, cannabidiol, and azithromycin, were tested at the beginning of the pandemic (Morris et al., 2020; Pereira et al., 2021) and have proven to be one of the least efficient strategies. Laboratory and patient data thus far do not support their use in the affected population. In practice, especially in our study, the use of some antifungals and antimalarials did not show promising results, and in the specific case of the antifungals, patients who used them were more likely to die. Another explanation for the high mortality associated with antifungal use in the present study could be that this drug was mostly prescribed to prevent or treat invasive fungal infections in the lung, such as COVID-19-associated pulmonary aspergillosis (CAPA). COVID-19 co-infections, such as CAPA, presented in 6.9% of patients admitted in the intensive care unit in previous studies (Salmanton-García et al., 2021). As with other drugs, randomized clinical trials are required to confirm or refute the experimental and observational results for each treatment described in the literature.

Ivermectin is a drug from the avermectin family that has antiparasitic activity against nematodes, scabies, and lice (Chen and Kubo, 2018; Kaur et al., 2021), stimulating the opening of chloride channels controlled by glutamic acid, leading to paralysis and death of these parasites. In addition, ivermectin also acts on gamma-aminobutyric acid-mediated chlorine channels and histamine- or PH-mediated chlorine channels (Martin et al., 2021). This drug also modulates these channels and receptors in humans, especially in the central nervous system; however, the impact of this modulation is still being studied worldwide (Martin et al., 2021). Even before the pandemic, ivermectin had been studied in the treatment of microorganisms, including viruses, bacteria, and protozoa, and the results so far have been promising (Mastrangelo et al., 2012; Wagstaff et al., 2012; Crump, 2017). In addition to its direct effect on ectoparasites and endoparasites, ivermectin modulates the recruitment of immune cells and the production of cytokines and chemokines to control inflammatory and immune processes. For example, ivermectin induces a protective Th1 immune response against protozoa of the genus Leishmania and increases the immune response and survival of mice infected with *Trypanosoma brucei* (Udensi and Fagbenro-Beyioku, 2012). This drug also increases the phagocytic and microbicidal activity of macrophages (Sajid et al., 2007; Omer et al., 2012) and suppresses mucus hypersecretion, IgE and IgG1 production, cell recruitment, and cytokine production in the lung in an experimental model with rabbits (Yan et al., 2011; Crump, 2017).

Regarding the virus that causes COVID-19, findings with ivermectin are controversial, and clinical trials are still being conducted worldwide. *In silico*, *in vitro*, and *in vivo* assays have shown sufficient potential for use in randomized clinical trials (Becker and Stadler, 2021; Kaur et al., 2021; Rajter et al., 2021). For example, ivermectin has demonstrated effects on clinical recovery, disease progression, mortality of COVID-19 patients (Emadi et al., 2020; Vallejos et al., 2020; Abd-Elsalam et al., 2021; Behera et al., 2021; Hellwig and Maia, 2021; Kaur et al., 2021; Popp et al., 2021). In our findings, prior use of ivermectin by some patients did not result in improvement and survival after hospitalization. Dosage, duration of use, or other information needed to track the use of this and other medications before admission could not be obtained or evaluated. In a study using a clinical design, the authors also did not observe improvements in the duration of symptoms in patients who used ivermective in relation to placebo patients (López-Medina et al., 2021). Added to this in another recent randomized clinical trial involving patients who had had symptoms of COVID-19 for up to 7 days and had at least one risk factor for disease progression were evaluated for ivermectin activity the authors reported that treatment with ivermectin did not result in a lower incidence of medical admission to a hospital due to progression of COVID-19 or of prolonged emergency department observation among outpatients with an early diagnosis of COVID-19 (Reis et al., 2022). Obviously, the discussion about the use or non-use of these and other drugs needs to be conclusively resolved as the usage of these drugs have surpassed scientific limits and entered political discussions in several countries distracting from the fight against the devastating COVID-19 pandemic.

Taken together, the data presented in this study suggest that the challenges to completely resolve this pandemic remain. Conversely, the epidemiological data demonstrated here also indicate some treatments and variables that should be considered for continued improvements in patient populations. It is worth noting that the older the patient, the use of antifungals has a more negative impact, and the use of anticoagulants has a more positive impact, in terms of the mortality, which increases the chance of life for hospitalized patients who are positive for COVID-19 infection.

## Conclusions

COVID-19 emerged recently, and new data and information about the disease are published daily. Given the amount and depth of information coming from so many new publications, our understanding of the COVID-19 disease and infection is evolving. In this study, age, presence of comorbidities, length of hospitalization, and drug treatment defined the highest or lowest mortality rates in patients. With these data, it is necessary to develop public policies to monitor patients and treatments and, if possible, outline the preventive and curative measures that best fit each patient.

## Data Availability Statement

The original contributions presented in the study are included in the article/supplementary material. Further inquiries can be directed to the corresponding author.

## Ethics Statement

The studies involving human participants were reviewed and approved by Approved by the Research Ethics Committee of the Hospital de Clínicas, Universidade Federal do Triângulo Mineiro (HC-UFTM) (approval number: 3.957.676). The patients/participants provided their written informed consent to participate in this study.

## Author Contributions

CO designed the experiments. BO, WR, DA, DS, LA, CD, TF-d-A, JC-M, RB, AB, AH, LP, FH, ML, LB, RT, MO, GB, FM, AO-S, IM, YF, GM, K-FP, HM-S, MS, and VR performed the experiments. CO and WR analyzed the data. BO, WR, DA, DS, LA, CD, TF-d-A, JC-M, RB, AB, AH, LP, FH, ML, LB, RT, MO, GB, FM, AO-S, IM, YF, GM, KF-P, HM-S, MS, VR, and CO wrote the manuscript. All authors contributed to the article and approved the submitted version.

## Funding

This work was supported by the Federal University of Triângulo Mineiro (UFTM), Fundação de Amparo à Pesquisa do Estado Minas Gerais (FAPEMIG), National Council for Scientific and Technological Development (CNPq), and Coordination for the Improvement of Higher Education Personnel (CAPES; Finance code 001).

## Conflict of Interest

The authors declare that the research was conducted in the absence of any commercial or financial relationships that could be construed as a potential conflict of interest.

## Publisher’s Note

All claims expressed in this article are solely those of the authors and do not necessarily represent those of their affiliated organizations, or those of the publisher, the editors and the reviewers. Any product that may be evaluated in this article, or claim that may be made by its manufacturer, is not guaranteed or endorsed by the publisher.

## Abbreviations

COVID, coronavirus disease; OR, odds ratio; CI, confidence interval.
